# The effects of executive functions on language control during Chinese-English emotional word code-switching

**DOI:** 10.3389/fpsyg.2023.1087513

**Published:** 2023-01-25

**Authors:** Jiao Zhang, Lin Fan

**Affiliations:** ^1^Faculty of Foreign Languages, Ningbo University, Ningbo, China; ^2^National Research Centre for Foreign Language Education, Beijing Foreign Studies University, Beijing, China

**Keywords:** EFs, language control, IC ability, emotional valence, emotional congruency, emotional word comprehension

## Abstract

Executive functions (EFs) have great impact on language control indexed by language switch costs during production-based language switching. Yet, how they influence language control during comprehension-based language switching between embodied first language (L1, Chinese) emotional words and less embodied second language (L2, English) emotional words is less understood. Employing an emotional priming paradigm, this study recruited Chinese-English bilinguals as participants, and used emotional faces and words as experimental materials to explore the effects of cool [i.e., inhibitory control ability, IC ability] and hot (i.e., emotional valence and emotional congruency) EFs on language switch costs (i.e., language control) during Chinese-English emotional word comprehension. The results showed larger language switch costs in the emotional congruent condition relative to emotional conflict condition, larger Chinese switch costs than English switch costs, and larger language switch costs for negative over positive emotional words in the emotional congruent condition. In addition, high-IC participants showed larger English switch costs for negative emotional words compared with low-IC participants. These results indicated that hot EF and the embodiment of language had an impact on both language control and the modulation of cool EF on language control, and that the components of hot EFs interacted and jointly affected language control during language switching.

## Introduction

Code-switching (also called language switching) refers to the alternation of two languages, dialects or varieties within a single discourse, sentence or constituent (Poplack, 1980). When it occurs, bilingual speakers’ non-target language is activated non-selectively and creates interference, thus they would rely on certain cognitive control mechanisms to select an appropriate language to communicate at a critical point and minimize the interference from the unintended language, which is known as “language control” (Abutalebi et al., 2008). Given the overlapping mechanisms (Declerck et al., 2017) and the common brain regions involved (Hernandez and Meschyan, 2006) in language control and executive functions (EF), the questions remain how bilinguals implement language control and whether EF, which refers to the psychological processes in the conscious control of thought and action and involves cool EF and hot EF^1^ (Zelazo and Müller, 2002), plays a role in language control processes. Cool EF associated with the dorsolateral prefrontal cortex (DL-PFC) is more likely to be elicited by relatively abstract, emotionally neutral materials; hot EF associated with orbitofrontal cortex (OFC) is more likely to be involved when the stimuli are characterized by high affective involvement or demand flexible appraisals of the affective significance (Zelazo and Müller, 2002: 455).

Empirical evidence from most production-based language switching studies substantiates that IC of cool EFs is the primary mechanism used in language control (e.g., Linck et al., 2012; Babcock and Vallesi, 2015; Liu et al., 2019, but see Costa and Santesteban, 2004 for exceptional cases), which is supported by the inhibitory control model (IC model) proposed by Green (1998). IC is a basic cognitive depression that facilitates task execution by preventing unrelated information from entering into the task or remaining in working memory (Harnishfeger and Bjorklund, 1993). According to IC model, in order to switch to a word of a particular language, the new schema and language tag must be activated and the previously activated schema and language tag must be suppressed (Wang et al., 2009). The IC process through schema and language tag suppression takes time and thus yields language switch costs indexed by the reaction time (RT) difference between language switch (the languages of at least two consecutive trials are different) and repetition (the languages of at least two consecutive trials are the same) trials. The magnitude of language switch costs has been taken as the main indicator of the difficulty of language control (e.g., Guo et al., 2013; Prior and Gollan, 2013; Liu et al., 2019, 2020). In view of the important role of IC mechanism in language control, researchers initiated the research on the effects of participants’ domain-general IC ability on language switch costs in bilingual language production (e.g., Linck et al., 2012; Liu et al., 2017; Chang et al., 2018; Liu et al., 2019). The results showed that participants with high-IC ability showed smaller language switch costs than those with low-IC ability, suggesting a nexus between participants’ domain-general IC ability and language switch costs in particular, and between their EFs and language control in general.

To reveal the mechanisms of language control in bilinguals, researchers also focus their attention on language control in bilingual visual word recognition. Different from the mechanisms of language control in spoken word production, language control in visual word recognition refers to the ability to understand the meaning of written words pertaining to a given language while reducing interference from the unrelated language (Mosca and de Bot, 2017). It is a crucial point of controversy over whether IC of cool EF is applied to comprehension-based language switching. Thomas and Allport (2000) proposed that there were two types of inhibition in language recognition tasks. One was the inhibition of language representations within the bilingual lexico-semantic system and the other was the inhibition of language task schema outside the bilingual lexicon. However, Macizo et al. (2012), Reynolds et al. (2016), and Struys et al. (2019) have proposed that the stimuli in bilingual recognition tasks are univalent, meaning that they do not generate response competition, so bilinguals do not have to rely on IC to solve the competition like production-based language switching. Liu et al. (2013) explored the effects of cognitive flexibility on both comprehension- and production-based language switching. The results indirectly showed that bilinguals’ IC ability played an important role in language switching of both speech production and visual word recognition. So far, the role of IC ability in comprehension-based language switching and the mechanisms of language control in bilingual language recognition remain unclear. Therefore, the first focus of this study is to explore the role of bilinguals’ domain-general IC ability of cool EF in comprehension-based bilingual language switching.

Zelazo and Müller (2002) have proposed that it is probably impossible to design a task that is a pure measure of hot or cool EF, and DL-PFC and OFC which are, respectively, responsible for cool EF and hot EF are parts of a single coordinated system and work together in a normal case. This suggests that cool and hot EFs may work together in the same task. However, available evidence for the effects of EFs on language control in language switching tasks has traditionally been based on cool EFs, and thus another major concern for this study involves the relationship between hot EFs and language control in bilingual language switching. Bond and Lai (1986) found that Cantonese-English bilinguals would switch into their second language (L2) while answering embarrassing questions in their first language (L1), suggesting that code-switching may exert its effects on emotion processing to serve an emotionally distancing function. In contrast, through immigrant parent–child interactions, Williams et al. (2020) found that facial emotion behavior had an immediate impact on code-switching, i.e., negative facial emotion predicted a higher frequency of bilingual code-switching in two directions and positive facial emotion predicted a lower frequency of code-switching in L2-L1 direction. Zhang et al. (2020) found that bilinguals showed longer response latencies and lower accuracy for Chinese-English negative emotional words compared to positive ones during Chinese-English emotional word code-switching, suggesting the important role of emotional valence in code-switching. Based on the above results, it is reasonable to conclude that previous studies have reached opposite or contradictory conclusions concerning the relationship between hot EFs and code-switching.

In order to unveil the underlying mechanisms between hot EFs and code-switching, the present study attempted to employ two types of emotional salient materials (i.e., emotional faces and emotional words with two dimensions of valence and arousal^2^) to explore the role of emotional valence (positive and negative) and emotional congruency (congruent and conflict) on language control during bilingual language switching with the emotional priming paradigm proposed by Fazio et al. (1986). In the emotional priming paradigm, emotional priming stimuli are used to elicit participants’ emotion to put them in a corresponding emotional state, then the emotional target stimuli are presented for them to finish a certain task. When the valences of emotional priming and target stimuli are different (emotional conflict condition), the RTs for the processing of emotional target stimuli are longer than those in the emotional congruent condition (the valences of emotional priming and target stimuli are the same), which is known as the emotional congruency effects or emotional priming effects (Haas et al., 2006; Yao and Wang, 2014; Başgöze et al., 2015). In our study, the emotional faces were used as emotional priming stimuli and the emotional words were used as emotional target stimuli. Participants were instructed to categorize the emotional valence of emotional words in both emotional congruent and conflict conditions. Their responses in the emotional conflict condition might be slower as compared with the emotional congruent condition in terms of the emotional congruency effects, resulting in larger language switch costs in the emotional conflict condition. Given the influence of language embodiment (due to language proficiency) on emotional word processing, this study would capitalize on positive and negative emotional words from two languages (i.e., L1 Chinese and L2 English) with different proficiency levels. For unbalanced late bilinguals, the less proficient L2 gets locked out of emotional contexts, thereby generating less embodied emotional words in the L2 (Sheikh and Titone, 2016). Conversely, the more proficient L1 can integrate the information from sensorimotor experience, emotional experiences and autobiographical memories, thus producing emotional words with higher embodiment in the L1 (Pavlenko, 2012). Based on the above embodiment difference between L1 and L2 emotional words, the dominant Chinese emotional words would have higher emotional activation than the weaker English emotional words. Therefore, participants need to allocate more resources to inhibit the higher activation of Chinese emotional words to facilitate the processing of English emotional words, and invest more cognitive processing resources to reactivate the previously suppressed Chinese than the previously suppressed English during language switching (Wang et al., 2009; Liu et al., 2019). According to these accounts, bilinguals might encounter larger difficulty in switching to L1 than to L2 and thus as a whole exhibit larger Chinese switch costs over English switch costs.

In addition to the respective impacts of hot EF and cool EF on language control during emotional word code-switching, this study also focused on the interactive effects of two EFs on language control. Zelazo and Müller (2002) proposed that OFC associated with hot EF typically developed earlier than DL-PFC associated with cool EF and came to be regulated by it, and they were parts of a single coordinated system and worked together in the normal case—even in a single situation, which indicates an intimate relationship between cool and hot EFs. According to Pessoa (2009), emotion has a great impact on human behavior and this impact crucially depends on how emotional stimuli affect the flow of EFs (the exact cool EF in Zelazo and Müller, 2002), such as directly convey emotional information to EF structures or direct prioritized attention toward strengthened sensory representations. The statements of Pessoa (2009) show the interplay between hot and cool EFs on human behavioral performance. The contribution of cool and hot EFs to decision-making (Séguin et al., 2007), academic achievement, learning related behaviors, and classroom behavior (O’Toole et al., 2020) can provide empirical evidence for the above indications. The findings of Başgöze et al. (2015) also lend support to the above indications. Employing an emotional word-face Stroop task to create emotional congruent and conflict trials with emotional words and faces, Başgöze et al. (2015) found interference related slowdown in emotional conflict trials for healthy participants and attributed it to the recruitment of inhibitory processes to remove the emotional conflict. Yet, how cool EF interacts with hot EF to affect bilinguals’ language control behavior during language switching is less understood. Hence, the interaction between the two types of EFs is taken into account in the current study to better reveal the impacts of EFs on language control during language switching. Taken together, this study attempted to adopt the emotional priming paradigm to explore the effects of cool (e.g., IC ability) and hot (e.g., emotional valence and emotional congruency) EFs on the language switch costs of Chinese-English emotional word comprehension to reveal the role of EFs in language control during this process. Specifically, the current study addressed whether IC ability, emotional valence, emotional congruency and the embodiment of language could impose effects on the language switch costs of Chinese-English emotional word comprehension and whether these factors could interact with each other. According to the above review of prior studies, we hypothesized that larger language switch costs may be observed for participants with high-IC ability over their low counterparts, for negative emotional words over positive emotional words, for emotional conflict condition over emotional congruent condition, as well as for embodied language (Chinese) over less embodied language (English). Moreover, it was also hypothesized that emotional valence and emotional congruency might influence the effects of IC ability on language switch costs, and these variables might interact with each other to jointly affect the language switch costs.

## Materials and methods

### Participants

The participants were 85 late Chinese-English (Chinese L1, English L2) bilinguals from Ningbo University in China [44 males; mean (M) age = 20.1 years; standard deviation (SD) = 1.3 years; range: 18–24 years]. They were right-handed and had normal or corrected-to-normal vision. All had passed College English Test Band 4 (CET-4), an official and well-established English test implemented by the Ministry of Education of the People’s Republic of China to measure Chinese non-English major college students’ English proficiency in writing, listening, reading and translating. Each participant was required to finish an informed consent form and a revised language history questionnaire originally designed by Li et al. (2006). The results of their questionnaires showed that they began learning their L1 from early childhood in the natural environment and their L2 at the age ranging from 7 to 13 years in the context of classroom. According to the theory of language embodiment put forth by Pavlenko (2005), these participants’ L1 Chinese are embodied due to the integration of phonological forms of words with the information from participants’ multiple sensory modalities, autobiographical memories and emotion, whereas their L2 English are less embodied due to the decontextualized nature of L2 classroom. The results of paired-samples *t*-tests showed that the proficiency of their Chinese listening, speaking, reading and writing and overall Chinese proficiency were significantly higher than their English counterparts (*p*s < 0.001; see Table 1).

**Table 1:** Means and SDs of the proficiency ratings for four skills of Chinese and English.

Skills	L1 (Chinese)	L2 (English)	*t*	*p*
Listening	6.6 (0.8)	3.3 (0.9)	27.014	0.000***
Speaking	6.4 (1.0)	3.2 (0.9)	25.375	0.000***
Reading	6.2 (1.2)	3.8 (0.9)	15.768	0.000***
Writing	5.7 (1.3)	3.3 (0.7)	15.932	0.000***
Overall proficiency	6.2 (0.9)	3.4 (0.6)	25.841	0.000***

***p < 0.001.

### Measure of IC ability and the grouping

The Simon task was administered to measure participants’ IC ability. In this task, trials began with a red fixation cross “+” presented in the center of the screen for 500 ms, followed by a filled red or blue square (2°× 2°, 2.1 cm in edge length) displayed at fixation, on the left of fixation or on the right of fixation. The square remained on the screen for 1,500 ms if no response was given, then a blank of 500 ms appeared. Half of the participants were informed to press the “F” key if the square appeared in red and the “J” key if the square appeared in blue. The other half of the participants were instructed the opposite. This task consisted of a block of 12 practice trials for participants to familiarize themselves with the experimental procedure, and 3 blocks of 360 experimental trials with 60 red and 60 blue square trials in each block (20 trials per location). Also, each block consisted of 40 congruent, 40 conflict and 40 neutral trials. In the congruent trials, the squares were presented on the same side of the reaction keys. In the conflict trials, the squares were displayed on the opposite side of the reaction keys. In the neutral trials, the squares appeared at fixation.

According to the median of the Simon costs as indexed by the RT difference between congruent and conflict trials in Simon task, the valid participants were divided into high (with smaller Simon costs) and low (with larger Simon costs) IC ability groups. The results of paired-samples *t*-tests showed shorter RTs [*t* (82) = −11.717, *p* < 0.001] and lower error rates (ERs) [*t* (82) = −3.578, *p* < 0.05] for participants with high-IC ability than those with low-IC ability, suggesting that the grouping is effective. Further analysis revealed no significant differences between the high- and low-IC participants in the age of acquisition (AoA), CET-4 score, length for studying both L1 and L2, four skills of L1 and four skills of L2 (all *p*s > 0.05). Also, no significant differences were observed in the overall L1 proficiency (High: *M* = 6.1, *SD* = 0.9; Low: *M* = 6.3, *SD* = 1.0), *t* (82) = −0.874, overall L2 proficiency (High: *M* = 3.5, *SD* = 0.6; Low: *M* = 3.3, *SD* = 0.5), *t* (82) = 0.979, as well as the overall L1 and L2 language proficiency (High: *M* = 4.8, *SD* = 0.6; Low: *M* = 4.8, *SD* = 0.6) between the high- and low-IC participants, *t* (82) = −0.203, *p*s > 0.05. Table 2 presents the two groups’ descriptive statistics of Chinese and English proficiency.

**Table 2:** Means and SDs of the AoA, CET scores, learning time and proficiency ratings of four language skills for high- and low-IC ability groups’ Chinese and English.

Manipulation variables	L1 (Chinese)				L2 (English)			
	High-IC	Low-IC	*t*	*p*	High-IC	Low-IC	*t*	*p*
AoA	–	–	–	–	8.9 (1.4)	9.2 (1.7)	−0.911	0.365
CET	–	–	–	–	504.5 (33.7)	500.4 (36.5)	0.541	0.590
Length	19.8 (1.1)	20.2 (1.3)	−1.524	0.131	10.9 (1.6)	11.0 (2.0)	−0.422	0.674
Listening	6.6 (0.7)	6.6 (0.9)	−0.274	0.785	3.4 (0.9)	3.2 (0.9)	0.979	0.330
Speaking	6.4 (0.9)	6.5 (1.0)	−0.574	0.568	3.4 (0.9)	3.1 (0.8)	1.436	0.155
Reading	6.0 (1.2)	6.3 (1.1)	−1.411	0.162	3.9 (1.0)	3.8 (0.8)	0.376	0.708
Writing	5.6 (1.4)	5.8 (1.3)	−0.654	0.515	3.2 (0.7)	3.3 (0.7)	−0.157	0.876

### Materials

The materials included emotional primes and targets. The primes were 40 negative (20 males and 20 females; valence range: 2.1–3.3; arousal range: 4.3–7.7) and 40 positive (20 males and 20 females; valence range: 5.2–7.0; arousal range: 5.0–8.0) emotional faces rated with a 9-point Likert scale, which were selected from Chinese Facial Affective Picture System (CFAPS-P, Gong et al., 2011) and displayed in a height of 8.4 cm and a width of 6.8 cm. The results of independent-samples *t*-tests showed a significant difference between the valence of positive and negative faces (*p* < 0.001), but their arousal showed an insignificant difference (*p* > 0.05). Additionally, neither the valence nor the arousal showed significant differences between male and female faces (*p*s > 0.05).

The targets were 40 English emotional words and their Chinese translation equivalents (i.e., 40 Chinese emotional words), with the former taken from the appendices of previous studies (e.g., Harris et al., 2003; Scott et al., 2009; Kazanas and Altarriba, 2016), *Affective Norms for English Words* (ANEW, Bradley and Lang, 1999), *The Book of Human Emotions* (Smith, 2016) and *Pocket Oxford English-Chinese Dictionary* (Soanes, 2009). Each language included 20 positive (valence range: 4.8–6.6; arousal range: 3.8–5.4) and 20 negative (valence range: 1.3–3.4; arousal range: 3.7–5.3) emotional words rated with a 7-point Likert scale (see Supplementary material). The results of independent-samples *t*-tests revealed no significant differences in the familiarity, frequency, abstractness, arousal and valence between Chinese and English emotional words (*p*s > 0.05) and in the familiarity, frequency, abstractness and arousal between positive and negative emotional words (*p*s > 0.05), yet the valence showed a significant difference between positive and negative emotional words (*p* < 0.001; see Table 3). As shown in Table 4, Chinese positive and negative emotional words had no significant differences in their stroke number, frequency, familiarity, arousal and abstractness (*p*s > 0.05), but their valence showed an obvious difference (*p* < 0.001). As for English positive and negative emotional words, there were no significant differences in their syllable number, letter number, frequency, familiarity, arousal and abstractness (*p*s > 0.05), but their valence revealed a significant difference (*p* < 0.05). In addition, no significant differences were observed in the word frequency, familiarity, abstractness, arousal and valence between Chinese and English positive emotional words, as well as in those variables between Chinese and English negative emotional words (*p*s > 0.05).

**Table 3:** Means and SDs of stimulus attributes for Chinese, English, positive and negative emotional words.

Manipulation variables	L1 (Chinese)	L2 (English)	*t*	Positive	Negative	*t*
Familiarity	6.2 (0.2)	6.2 (0.6)	0.148	6.3 (0.5)	6.1 (0.5)	1.379
Frequency	27.0 (23.4)	18.8 (15.7)	1.831	25.9 (23.6)	19.9 (15.8)	1.328
Abstractness	3.6 (0.3)	3.6 (0.3)	−0.235	3.6 (0.4)	3.6 (0.3)	0.364
Arousal	4.8 (0.4)	4.7 (0.4)	0.781	4.7 (0.4)	4.7 (0.4)	−0.148
Valence	3.9 (1.8)	4.0 (1.7)	−0.129	5.6 (0.5)	2.3 (0.5)	31.744

**Table 4:** Means and SDs of stimulus attributes for Chinese and English positive and negative emotional words.

Manipulation variables	L1 (Chinese)				L2 (English)			
	Positive	Negative	*t*	*p*	Positive	Negative	*t*	*p*
Syllables	–	–	–	–	2.5 (0.9)	2.1 (0.6)	1.633	0.112
Letters	–	–	–	–	7.6 (1.9)	7.0 (1.9)	1.000	0.324
Strokes	18.8 (4.9)	17.5 (3.8)	0.940	0.353	–	–	–	–
Frequency	30.5 (28.0)	23.5 (17.6)	0.942	0.353	21.3 (17.7)	16.3 (13.3)	1.002	0.323
Familiarity	6.3 (0.2)	6.1 (0.2)	1.585	0.121	6.3 (0.6)	6.1 (0.6)	0.862	0.394
Arousal	4.8 (0.5)	4.8 (0.4)	−0.287	0.776	4.7 (0.4)	4.7 (0.4)	0.091	0.928
Abstractness	3.6 (0.4)	3.6 (0.2)	0.363	0.719	3.6 (0.3)	3.6 (0.4)	0.152	0.880
Valence	5.7 (0.5)	2.2 (0.5)	21.977	0.000	5.6 (0.4)	2.4 (0.5)	22.977	0.000

### Design

A 2 (IC ability: high and low) × 2 (Language: Chinese and English) × 2 (Emotional valence: positive and negative) × 2 (Emotional congruency: congruent and conflict) analysis of variance (ANOVA) was employed, with IC ability as a between-subjects independent variable, language, emotional valence and emotional congruency as within-subjects independent variables, and language switch costs indicated by mean RTs and ERs as dependent variables.

### Procedures and tasks

**Figure 1:**
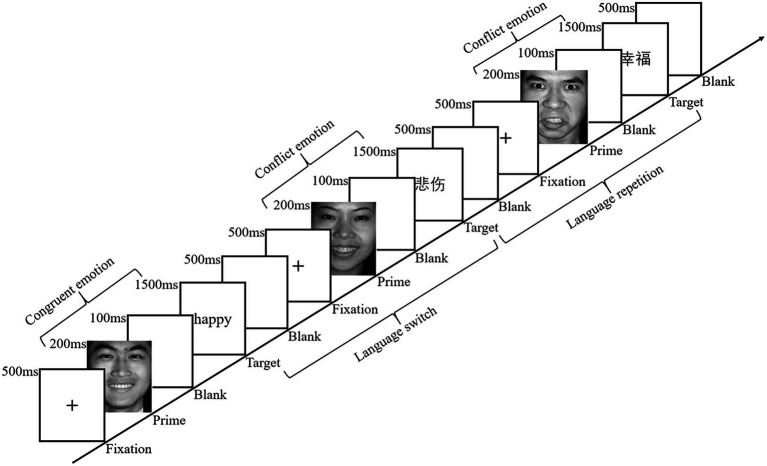
Flowchart of three experimental trials. Reproduced with permission from Yaojia Luo. Facial images reproduced with permission from Gong et al. (2011).

The experiment employing an emotional priming paradigm was performed individually on a desktop computer with a 20-inch monitor in the laboratory of Ningbo University in China. In the experiment, E-prime 2.0 was used to present stimuli and collect data. Participants were seated in front of the screen at a distance of approximately 60 cm. They were instructed to categorize the valence of emotional words in the valence categorization task (*cf*., Houwer et al., 2001) including 12 practice trials and 4 blocks of 320 experimental trials. Each trial started with a red fixation cross (+) presented in the center of the screen for 500 ms, followed by a 200 ms emotional face as a prime to evoke participants’ emotion and then a 100 ms blank screen. After that, a Chinese or English emotional word as a target appeared for 1,500 ms until a response was given or 1,500 ms had elapsed, then a blank screen of 500 ms appeared (see Figure 1). Participants were informed to press the F-key on the keyboard with the left index finger if the word was positive and press the J-key on the keyboard with the right index finger if the word was negative. The valence-response key association was counterbalanced across participants.

The experimental trials were divided into previous and current trials according to the sequence. In terms of this division and the valence of emotional words, the overall trials can be categorized into four types of 80 trials each: positive–positive, positive–negative, negative–negative and negative–positive. Based on the emotional congruency of priming and target stimuli, 4 sequence relation types of 80 trials each were also constructed: congruent-congruent, congruent-conflict, conflict-congruent, conflict-conflict. The repetition and switch of both language and emotional valence of emotional word were balanced. In all, there were 40 language repetition positive trials, language repetition negative trials, language switch positive trials, language switch negative trials in both L1 and L2. The experiment lasted approximately 20 min. Short breaks were given between blocks.

## Results

One participant’s data was excluded due to accuracy rate lower than 80% in the valence categorization task. Data from the first three trials, trials with wrong responses (7.4%), RTs less than 200 ms (0.1%) and exceeding *M* ± 3*SD* (1.7%) in the valence categorization task were discarded. The remaining data were analyzed by SPSS 17.0. A 2 × 2 × 2 × 2 repeated measures ANOVA with IC ability (high vs. low), language (Chinese vs. English), emotional valence (positive vs. negative) and emotional congruency (congruent vs. conflict) was performed. Due to space constraints, the results of insignificant interactions were not reported. Table 5 presented the descriptive statistics of the two groups’ language switch costs in different emotional conditions.

**Table 5:** Descriptive statistics of high- and low-IC ability groups’ language switch costs in different emotional conditions.

Conditions	Switch costs	High-IC				Low-IC			
		L1 (Chinese)		L2 (English)		L1 (Chinese)		L2 (English)	
		Positive	Negative	Positive	Negative	Positive	Negative	Positive	Negative
Congruent	RTs	27.5 (59.0)	27.7 (46.7)	0.7 (37.8)	34.4 (54.7)	24.5 (38.9)	19.4 (48.3)	−2.5 (46.9)	8.3 (53.6)
	ERs	−1.0 (5.1)	0.1 (8.5)	−0.8 (6.6)	−0.8 (10.5)	−2.4 (6.6)	−0.2 (7.2)	−0.6 (8.1)	−1.4 (10.4)
Conflict	RTs	35.2 (33.0)	−10.1 (57.2)	−9.7 (41.2)	12.8 (56.3)	26.3 (31.4)	8.2 (45.7)	−12.9 (57.3)	2.1 (56.1)
	ERs	0.2 (4.8)	−0.1 (7.2)	−1.4 (7.9)	−2.3 (10.1)	0.5 (5.9)	−1.1 (7.7)	−3.5 (8.9)	−0.2 (10.8)

The RT data analysis yielded significant main effects of emotional congruency, *F* (1, 82) = 9.943, *p* < 0.05, *η*_p_^2^ = 0.108, and language, *F* (1, 82) = 16.495, *p* < 0.001, *η*_p_^2^ = 0.167, with larger language switch costs in the emotional congruent condition relative to emotional conflict condition and larger Chinese switch costs than English switch costs. No significant main effects of IC ability and emotional valence were observed (*p*s > 0.05). The interaction between emotional congruency and emotional valence was significant, *F* (1, 82) = 4.541, *p* < 0.05, *η*_p_^2^ = 0.052. Simple effect analysis revealed smaller language switch costs for negative emotional words in the emotional conflict condition as opposed to the emotional congruent condition (*p* < 0.05), and larger language switch costs for negative emotional words relative to positive ones in the emotional congruent condition (*p* = 0.080). Furthermore, there was an emotional valence by language interaction, *F* (1, 82) = 24.361, *p* < 0.001, *η*_p_^2^ = 0.229. Simple effect analysis showed larger Chinese switch costs for positive emotional words compared to negative ones and opposite English switch cost pattern for negative and positive emotional words (*p*s < 0.05). In addition, participants’ Chinese switch costs were larger than their English switch costs for positive emotional words (*p* < 0.001).

The three-way interaction among emotional congruency, emotional valence and language reached significance, *F* (1, 82) = 5.188, *p* < 0.05, *η*_p_^2^ = 0.060. Simple simple effect analysis revealed larger Chinese switch costs for negative emotional words in the emotional congruent condition relative to emotional conflict condition, and larger Chinese switch costs for positive relative to negative emotional words in the emotional conflict condition (*p*s < 0.05). Moreover, participants’ English switch costs for negative emotional words were not only larger in the emotional congruent condition relative to emotional conflict condition (*p* = 0.058), but also larger than those for positive emotional words in both emotional congruent and conflict conditions (*p*s < 0.05), and their Chinese switch costs were larger than their English switch costs for positive emotional words in both emotional congruent and conflict conditions (*p*s < 0.001). Notably, the interaction among IC ability, emotional valence and language was marginally significant, *F* (1, 82) = 2.940, *p* = 0.090, *η*_p_^2^ = 0.035. Simple simple effect analysis indicated that participants with high-IC ability displayed larger English switch costs for negative emotional words than those with low-IC ability (*p* = 0.060) and larger Chinese switch costs for positive relative to negative emotional words (*p* < 0.05). Additionally, high-IC participants displayed larger English switch costs for negative emotional words relative to positive ones (*p* < 0.05) and smaller Chinese switch costs relative to English switch costs for negative emotional words (*p* = 0.085). As for positive emotional words, the two groups’ Chinese switch costs were significantly larger than their English switch costs (*p*s < 0.001).

The ER data analysis revealed that none of the main effects reached significance (all *p*s > 0.05). The three-way interaction among emotional congruency, emotional valence and language was marginally significant, *F* (1, 82) = 3.306, *p* = 0.073, *η*_p_^2^ = 0.039. Simple simple effect analysis showed smaller Chinese switch costs for positive emotional words in the emotional congruent condition relative to emotional conflict condition and smaller English switch costs than Chinese switch costs for positive emotional words in the emotional conflict condition (*p*s < 0.05).

## Discussion

The current study aimed to explore the impacts of cool and hot EFs on language control during comprehension-based language switching between embodied Chinese emotional words and less embodied English emotional words. The results showed larger Chinese switch costs than English switch costs and larger language switch costs in the emotional congruent condition relative to the emotional conflict condition, indicating that the embodiment of language and the emotional congruency eliciting the involvement of hot EF can modulate the language control during Chinese-English emotional word code-switching. These findings are in line with our prediction for the role of the embodiment of language, but in contrast to our prediction for the role of emotional congruency. In the emotional conflict condition, the occurrence of conflict can not only trigger participants’ adjustment mechanism and improve their cognitive control level in the same conflict task based on the emotion adaptation effect in conflict monitor theory proposed by Gratton et al. (1992), but also lead to the compensatory adjustments in perceptual selection, which in turn serves to alleviate conflict (Berlyne, 1960). Hence, participants’ language switch costs in the emotional conflict condition were smaller. Another possible reason is that the emotional congruent and conflict conditions may trigger participants’ appetitive and aversive motivational systems, respectively. Emotional conflict, as a kind of aversive signal (Dreisbach and Fischer, 2012; Fritz and Dreisbach, 2013), could trigger participants’ aversive motivational system and capture their attention easily and automatically (Gawronski et al., 2005), resulting in less cognitive processing resources invested to avoid the cognitive loss in the emotional conflict condition. However, in the emotional congruent condition, driven by the appetitive motivational system, participants are more inclined to take a risk and invest more cognitive processing resources to achieve better performance. This observation in our study contrasts the finding of Liu et al. (2019), which reported larger language switch costs for the conflict processing context over the congruent processing context. These divergent research findings may be attributed to the different manipulation of the congruent and conflict conditions in the two studies. Specifically, compared to the non-emotional conditions in Liu et al. (2019), the congruent and conflict conditions in our study were emotional, and congruency and emotions could jointly affect the language switch costs, thus modulating the language switch cost pattern of non-emotional conditions.

However, the insignificant difference between the language switch costs of positive and negative emotional words in our study suggested that emotional valence which can induce the involvement of hot EF did not have a significant impact on language control during Chinese-English emotional word comprehension. This finding was inconsistent with that of the first experiment of Zhang (2020), which reported larger language switch costs for positive emotional words than negative emotional words. One possible explanation for the disparate findings is the cognitive task load elicited by the emotional priming stimuli (i.e., emotional faces). In the emotional priming paradigm, the emotions displayed by emotional faces can be recognized automatically (see Hanley et al., 1990), thus preferentially occupying participants’ cognitive resources and leading to insufficient cognitive resources for the valence categorization task. Hence, no significant difference was observed between the language switch costs of positive and negative emotional words. This discrepancy may also be due to the modulating effects of emotional congruency on emotional valence in this study. Different from the processing in the first experiment of Zhang (2020), participants in our study should not only resort to IC ability of cool EFs for language switching, but also need to rely on hot EF to fulfill the valence categorization task and resolve the emotional conflict induced by the emotional priming paradigm. Therefore, part of the resources used for categorizing the valence of emotional words and switching languages in Zhang (2020), especially the resources in the hot emotional system, should be diverted to solve the emotional conflict, resulting in limited resources used for the valence categorization task and then insignificant difference between the language switch costs of positive and negative emotional words. This possible explanation was supported by the result of insignificant difference between the language switch costs of positive and negative emotional words in the emotional conflict condition.

Meanwhile, we also found smaller language switch costs for negative emotional words in the emotional conflict condition as opposed to the emotional congruent condition. van Steenbergen et al. (2009, 2010) found that emotions can regulate conflict-driven control. Specifically, positive emotion can offset the negative emotion elicited by conflict and reduce participants’ conflict-driven cognitive control, thus lessening the conflict adaptation effect; whereas negative emotion can enhance participants’ conflict-driven cognitive control, which in turn generates larger conflict adaptation effect, thus resulting in smaller language switch costs in the emotional conflict condition. This study also found larger language switch costs for negative over positive emotional words in the emotional congruent condition. A possible reason is that compared with conflict as an aversive signal (Dreisbach and Fischer, 2012; Fritz and Dreisbach, 2013), congruent emotion may be experienced as a pleasant signal which can enhance the emotional intensity of positive words and promote task switching (Wang and Guo, 2008), reducing the language switch costs. Another possible reason is that positive emotion can enhance cognitive flexibility (Isen and Daubman, 1984; Isen et al., 1992), which regulates the cognitive control by reducing the costs of information retention in task (Dreisbach, 2006), leading to smaller language switch costs for positive emotional words. The above results not only indicated that emotional congruency could modulate the effects of emotional valence on participants’ language control, but also indicated that different emotional information of hot EFs could interact and jointly affect participants’ language control during language switching.

Apart from the role of hot EFs, this study also concerned the impact of cool EF on language control. The insignificant main effect of IC ability in this study suggest that cool EF did not have a significant impact on language control during Chinese-English emotional word comprehension. This finding contrasts with that found in previous production-based language switching studies (Linck et al., 2012; Liu et al., 2014; Chang et al., 2018; Liu et al., 2019), reporting larger language switch costs for low-IC group over high-IC group. This discrepancy corroborated the different switching processes between comprehension- and production-based language switching proposed by other researchers (e.g., Qi et al., 2010; Reynolds et al., 2016; Struys et al., 2019). One possibility for this discrepancy lies in the distinct switching processes between language comprehension and production. In comprehension-based language switching, the stimuli are coded in a specific language (i.e., univalent), no response competition is generated, thus resorting to domain-specific control mechanism of language activation during language switching (Struys et al., 2019). In contrast, the stimuli in language production are bivalent which can elicit two responses, thus generating response competition. In this sense, participants should deploy domain-general IC mechanism to suppress the competition during language switching (Reynolds et al., 2016). Another plausible reason for this difference is whether the modulation of emotional information (hot EFs) occurs during language switching. Specifically, emotional stimuli were used in the current study and the conflict elicited in the language switching processes was emotional conflict, which may regulate participants’ language control during language switching, thus affecting the relationship between their IC ability and language control. However, non-emotional stimuli were employed in previous studies, thus evoking cognitive conflict without emotional information. Therefore, it might have little regulatory effect on language control, nor did it affect the relationship between participants’ IC ability and language control during the language switching processes.

However, it is worth noting that the interaction among IC ability, emotional valence and language was marginally significant. High-IC participants showed larger English switch costs for negative emotional words compared to low-IC participants. This finding can be attributed to high-IC participants’ longer RTs for negative emotional words on English switch trials, which was derived from the data analysis of the two groups’ RTs in English repetition and switch trials for negative emotional words. Pavlenko (2012) posited that the decreased automaticity of emotional processing in L2 on account of less embodied cognition can reduce interference effects and lower electrodermal reactivity to negative emotional stimuli, indicating the attenuated activation of L2 negative emotional words. With regard to English switch trials for negative emotional words, the attenuated activation of L2 negative emotional words may occupy less processing resources and retain more processing resources to switch languages for participants with both high- and low-IC ability. In addition, there was higher inhibition of English in the previous trials for high-IC participants over their low counterparts, thus it took longer for high-IC participants to overcome the inhibition. This result suggests that emotional valence can modulate the impact of IC ability on language control during language switching processes.

## Conclusion

This study adopted the emotional priming paradigm to explore the effects of both hot and cool EFs on language control during Chinese-English emotional word comprehension. The findings showed that (1) emotional congruency and the embodiment of language could modulate the language control; (2) emotional congruency and emotional valence interacted and jointly affected the language control; and (3) emotional valence could modulate the impact of IC ability on the language control. These results reveal for the first time that hot and cool EFs concurrently and interactively exert their influence on language control during Chinese-English emotional word comprehension. As mentioned previously, cool EFs include other cognitive skills besides IC ability, such as working memory and cognitive flexibility. Future studies exploring the effects of cool EFs on language control can involve more cognitive skills. Moreover, the primes and targets in the present study only include positively and negatively valenced stimuli, further studies on the effects of hot EFs on language control can use neutral faces and words to constitute a baseline condition, and better reveal the role of hot EFs through the comparative results between different valences.

## Data availability statement

The original contributions presented in the study are included in the article/Supplementary material, further inquiries can be directed to the corresponding author.

## Ethics statement

The studies involving human participants were reviewed and approved by the Ethics Committee of Ningbo University. The participants provided their written informed consent to participate in this study.

## Author contributions

JZ: conceptualization, data collection, formal analysis, investigation, methodology, resources, visualization, and writing – original draft. LF: conceptualization, investigation, methodology, project administration, and supervision. All authors contributed to the article and approved the submitted version.

## Funding

This work was supported by the Fundamental Research Funds for the Provincial Universities of Zhejiang (SJWY2022010), the General Scientific Research Project of Zhejiang Provincial Education Department (Y202249103), and the Double First-Class Disciplines Major Project of Beijing Foreign Studies University (2022SYLZD008).

## Conflict of interest

The authors declare that the research was conducted in the absence of any commercial or financial relationships that could be construed as a potential conflict of interest.

## Publisher’s note

All claims expressed in this article are solely those of the authors and do not necessarily represent those of their affiliated organizations, or those of the publisher, the editors and the reviewers. Any product that may be evaluated in this article, or claim that may be made by its manufacturer, is not guaranteed or endorsed by the publisher.

## Footnotes

^1^Cool EF denotes the more purely cognitive skills like inhibitory control, working memory and cognitive flexibility. Take inhibitory control for example, this type of cool EF can be induced by the location conflict between squares and reaction keys in the Simon task of this study. In contrast, hot EF refers to the more affective aspects of EF, which can be elicited by emotional words like "happy"/"sad" and emotional faces with emotional expressions like "glad"/"sorrowful."

^2^Valence is a bipolar dimension which classifies emotions into positive and negative, whereas arousal is a unipolar dimension that denotes the level of emotional activation in appetitive or aversive motivation system (Lang et al., 1997).
