# Corrigendum: The effects of executive functions on language control during Chinese-English emotional word code-switching

**DOI:** 10.3389/fpsyg.2024.1411095

**Published:** 2024-04-19

**Authors:** Jiao Zhang, Lin Fan

**Affiliations:** ^1^Faculty of Foreign Languages, Ningbo University, Ningbo, China; ^2^National Research Centre for Foreign Language Education, Beijing Foreign Studies University, Beijing, China

**Keywords:** EFs, language control, IC ability, emotional valence, emotional congruency, emotional word comprehension

In the published article, the reference for O'Toole et al., 2020 was incorrectly written as “O'Toole, S. E., Monks, C. P., Tsermentseli, S., and Rix, K. (2020). The contribution of cool and hot executive function to academic achievement, learning related behaviours, and classroom behaviour, early child dev. *Care* 190, 806-821. doi: 10.1080/03004430.2018.1494595”. It should be “O'Toole, S. E., Monks, C. P., Tsermentseli, S., and Rix, K. (2020). The contribution of cool and hot executive function to academic achievement, learning-related behaviours, and classroom behaviour. *Early Child Dev. Care* 190, 806–821. doi: 10.1080/03004430.2018.1494595”.

Additionally, in the published article, there was an error in the use of the term “switch costs”. The term “switch costs” should be removed from the text outlined below.

A correction has been made to *Materials and Methods, Design, Paragraph 1*. This sentence previously stated:

“A 2 (IC ability: high and low) × 2 (Language switch costs: Chinese and English) × 2 (Emotional valence: positive and negative) × 2 (Emotional congruency: congruent and conflict) analysis of variance (ANOVA) was employed, with IC ability as a between-subjects independent variable, language, emotional valence and emotional congruency as within-subjects independent variables, and language switch costs of mean RTs and ERs as dependent variables.”

The corrected sentence appears below:

“A 2 (IC ability: high and low) × 2 (Language: Chinese and English) × 2 (Emotional valence: positive and negative) × 2 (Emotional congruency: congruent and conflict) analysis of variance (ANOVA) was employed, with IC ability as a between-subjects independent variable, language, emotional valence and emotional congruency as within-subjects independent variables, and language switch costs indicated by mean RTs and ERs as dependent variables.”

A correction has also been made to *Results, Paragraph 1*. This sentence previously stated:

“A repeated measure of 2 × 2 × 2 × 2 ANOVA with IC ability (high vs. low), language switch costs (Chinese vs. English), emotional valence (positive vs. negative) and emotional congruency (congruent vs. conflict) was performed.”

The corrected sentence appears below:

“A 2 × 2 × 2 × 2 repeated measures ANOVA with IC ability (high vs. low), language (Chinese vs. English), emotional valence (positive vs. negative) and emotional congruency (congruent vs. conflict) was performed.”

The authors apologize for these errors and state that they do not change the scientific conclusions of the article in any way. The original article has been updated.

