# Supplementary figures and images for: The effects of executive functions on language control during Chinese-English emotional word code-switching
Source: Front Psychol. 2023 Jan 25;14:1087513. doi: 10.3389/fpsyg.2023.1087513 (PMC9905722; doi:10.3389/fpsyg.2023.1087513)

Supplementary Material

# English and Chinese emotional words


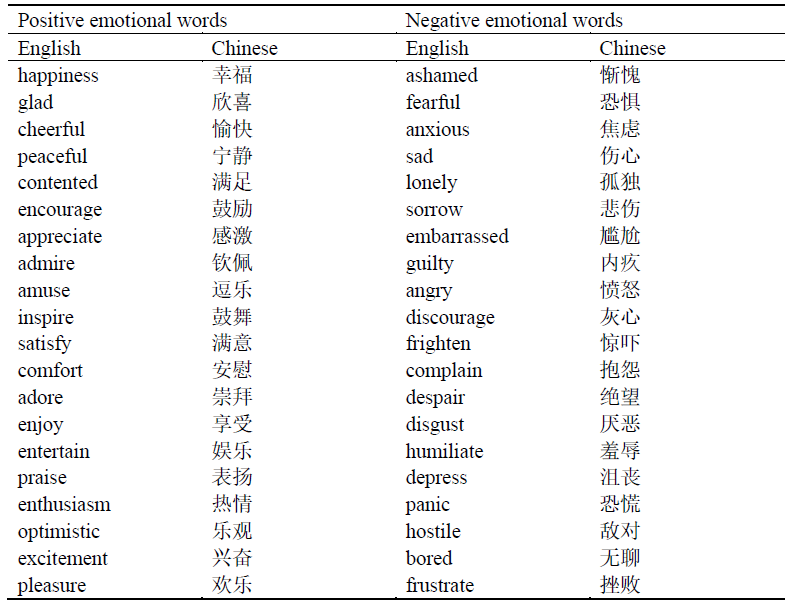

Supplement: Supplementary file 1 [file Table_1.DOCX]
